# Access to eye care during the COVID-19 pandemic, India

**DOI:** 10.2471/BLT.21.286368

**Published:** 2021-12-02

**Authors:** Janani Muralikrishnan, Josephine S Christy, Kavitha Srinivasan, Ganesh-Babu B Subburaman, Aakriti Garg Shukla, Rengaraj Venkatesh, Thulasiraj D Ravilla

**Affiliations:** a Department of Paediatrics, Aravind Eye Hospital, Pondicherry, India.; b Department of Cornea and Refractive Services, Aravind Eye Hospital, Pondicherry, India.; c Department of Glaucoma, Aravind Eye Hospital, Pondicherry, India.; d Department of Central Operations, Aravind Eye Care System, Madurai, India.; e Glaucoma Service, Wills Eye Hospital, Philadelphia, United States of America.; f Lions Aravind Institute of Community Ophthalmology, Aravind Eye Care System, 72 Kuruvukaran Salai, Annanagar, Madurai, Tamil Nadu, 625020, India.

## Abstract

**Objective:**

To study the impact of the coronavirus disease 2019 (COVID-19) pandemic on outpatient visits to eye care facilities in south India.

**Methods:**

We used data on 7.69 million outpatient visits to primary (i.e. vision centres), secondary and tertiary Aravind Eye Care System’s centres between January 2019 and June 2021. We compared outpatient numbers and outpatients’ age and sex between the pandemic period and the pre-pandemic period in 2019 for all centres, whereas vision and ophthalmic assessments were compared for vision centres only.

**Findings:**

During the first wave, the number of outpatient visits at tertiary, secondary and vison centres was 39% (647 968/1 656 296), 60% (170 934/283 176) and 73% (180 502/246 282) respectively, of 2019 levels. During the second wave, outpatient visits at tertiary, secondary and vision centres were 54% (385 092/710 949), 73% (88 383/121 739) and 79% (121 993/154 007), respectively, of 2019 levels. The proportion of outpatients who were female or younger than 20 years or older than 60 years was significantly lower during the first and second waves than in 2019 (*P* < 0.0001 for all). The proportion of outpatients whose worse eye vision was poorer than 5/60 or who required referral was significantly higher (*P* < 0.0001 for both).

**Conclusion:**

Restrictive measurements led to declines in outpatient visits, however the decline was less at secondary and vision centres than at tertiary centres. Easy access to specialized ophthalmic care via telemedicine and the relative proximity of these centres to communities helped reduce barriers to access.

## Résumé

**Objectif:**

Étudier l'impact de la pandémie de maladie à coronavirus 2019 (COVID-19) sur les consultations externes auprès des établissements de soins ophtalmologiques dans le sud de l'Inde.

**Méthodes:**

Nous avons utilisé les données concernant 7,69 millions de consultations externes réalisées dans les centres de soins primaires (cliniques de la vision), secondaires et tertiaires du réseau Aravind Eye Care System, entre janvier 2019 et juin 2021. Nous avons ensuite comparé le nombre de patients, leur âge et leur sexe entre la période marquée par la pandémie et celle précédant la pandémie en 2019 pour tous les centres, tandis que les évaluations ophtalmiques et examens de la vue n'ont été comparés que pour les cliniques de la vision.

**Résultats:**

Au cours de la première vague, le nombre de patients aux consultations externes dans les centres de soins tertiaires, secondaires et les cliniques de la vision s'élevait respectivement à 39% (647 968/1 656 296), 60% (170 934/283 176) et 73% (180 502/246 282) par rapport aux niveaux de 2019. Durant la deuxième vague, les consultations externes dans les centres de soins tertiaires, secondaires et les cliniques de la vision ont respectivement atteint 54% (385 092/710 949), 73% (88 383/121 739) et 79% (121 993/154 007) de leur niveau de 2019. Le pourcentage de patients de sexe féminin, âgés de moins de 20 ans ou de plus de 60 ans était nettement moins important pendant les première et deuxième vagues qu'en 2019 (*P* < 0,0001 pour l'ensemble de ces catégories). Enfin, le pourcentage de patients dont la vue était moins bonne que 5/60 ou qui avaient besoin d'être renvoyés vers un spécialiste avait considérablement augmenté (*P* < 0,0001 dans les deux cas).

**Conclusion:**

Les mesures de restriction ont entraîné un déclin des consultations externes. Néanmoins, ce déclin s'est révélé inférieur dans les cliniques de la vision et les centres de soins secondaires que dans les centres tertiaires. Un accès simplifié aux soins ophtalmiques spécialisés grâce à la télémédecine ainsi que la relative proximité de ces centres avec les communautés ont permis d'éliminer certains obstacles.

## Resumen

**Objetivo:**

Estudiar los efectos de la pandemia de la coronavirosis de 2019 (COVID-19) en las consultas externas de los centros de atención oftalmológica del sur de la India.

**Métodos:**

Se utilizaron los datos de 7,69 millones de consultas externas en centros de atención primaria (por ejemplo, centros oftalmológicos), secundaria y terciaria de la red Aravind Eye Care System, entre enero de 2019 y junio de 2021. Se comparó el número de pacientes ambulatorios, así como su edad y sexo, entre el periodo de la pandemia y el periodo previo a la pandemia en 2019 para todos los centros, mientras que las valoraciones de la visión y oftalmológicas se compararon solo para los centros oftalmológicos.

**Resultados:**

Durante la primera oleada, el número de consultas externas en los centros de atención terciaria, secundaria y oftalmológica fue del 39 % (647 968/1 656 296), el 60 % (170 934/283 176) y el 73 % (180 502/246 282), respectivamente, en relación con los niveles de 2019. Durante la segunda oleada, las consultas externas en los centros de atención terciaria, secundaria y oftalmológica fueron del 54 % (385 092/710 949), el 73 % (88 383/121 739) y el 79 % (121 993/154 007), respectivamente, en relación con los niveles de 2019. El porcentaje de pacientes ambulatorios que eran mujeres o menores de 20 años o mayores de 60 años fue muy inferior durante la primera y la segunda oleada que en 2019 (*p* < 0,0001 para todos). Asimismo, el porcentaje de pacientes ambulatorios que tenían una visión peor que 5/60 o que necesitaban ser derivados fue mucho mayor (*p* < 0,0001 para ambos).

**Conclusión:**

Las medidas restrictivas provocaron un descenso de las consultas externas, aunque el descenso fue menor en los centros de atención secundaria y oftalmológica que en los centros de atención terciaria. El fácil acceso a la atención oftalmológica especializada a través de la telemedicina y la relativa cercanía de estos centros a las localidades contribuyeron a reducir las barreras de acceso.

## ملخص

**الغرض:**

دراسة تأثير جائحة مرض فيروس كورونا 2019 (كوفيد 19) على زيارات المرضى الخارجيين لمرافق رعاية العيون في جنوب الهند.

**الطريقة:**

استخدمنا بيانات عن 7.69 مليون زيارة للمرضى الخارجيين إلى مراكز نظام أرافيند للعناية الأولية والثانوية والثالثية للعيون (أي مراكز الرؤية)، بين يناير/كانون ثاني 2019 ويونيو/حزيران 2021. قارنا بين أعداد المرضى الخارجيين وأعمارهم وجنسهم، في فترة الوباء وفترة ما قبل الوباء في عام 2019 لجميع المراكز، حيث تمت مقارنة تقييمات الرؤية والعيون لمراكز الرؤية فقط.

**النتائج:**

خلال الموجة الأولى، كان عدد زيارات العيادات الخارجية في مراكز الرؤية الثالثية والثانوية 39% (647968/1656296)، و60% (170934/283176)، و73% (180502/246282) على التوالي، من مستويات عام 2019. خلال الموجة الثانية، كان عدد زيارات العيادات الخارجية في مراكز الرؤية الثالثية والثانوي 54% (385092/710949)، و73% (88383/121739)، و79% (121993/154007)، على التوالي، من مستويات عام 2019 . كانت نسبة المرضى الخارجيين من الإناث، أو الذين تقل أعمارهم عن 20 عامًا، أو أكبر من 60 عامًا، أقل بشكل ملحوظ خلال الموجتين الأولى والثانية، منها في عام 2019 (نسبة الاحتمال أقل من 0.0001 للجميع). كانت نسبة المرضى الخارجيين الذين كانت رؤية العيون لديهم أسوأ من 5/60، أو الذين كانوا بحاجة إلى إحالة، أعلى بشكل ملحوظ (نسبة الاحتمال أقل من 0.0001 لكليهما).

**الاستنتاج:**

أدت القياسات المقيدة إلى انخفاضات في زيارات المرضى للعيادات الخارجية، إلا إن الانخفاض كان أقل في المراكز الثانوية ومراكز الرؤية مقارنة بالمراكز الثالثية. ساعد الوصول السهل إلى خدمات رعاية العيون المتخصصة عن طريق العلاج الطبي عن بعد، والقرب النسبي لهذه المراكز من المجتمعات المحلية، في تقليل الحواجز التي تحول دون الوصول لهذه الخدمات.

## 摘要

**目的:**

旨在研究新型冠状病毒肺炎（新冠肺炎）疫情对印度南部眼部护理机构门诊就诊情况的影响。

**方法:**

我们使用了 2019 年 1 月至 2021 年 6 月期间初级（即视力中心）、二级和三级亚拉文眼科关爱中心 (Aravind Eye Care System) 的 769 万次门诊就诊相关数据。我们比较了 2019 年所有中心在疫情期间和疫情开始前的门诊人数及门诊患者的年龄和性别，但仅对视力中心的视力和眼科评估进行了比较。

**结果:**

在第一波期间，三级、二级和视力中心的门诊就诊人数分别占 2019 年总人数的 39% (647,968/1,656,296)、60% (170,934/283,176) 和 73% (180,502/246,282)。在第二波期间，三级、二级和视力中心的门诊就诊率分别占 2019 年总水平的 54% (385,092/710,949)、73% (88,383/121,739) 和 79% (121,993/154,007)。在第一波和第二波期间，女性或小于 20 岁或大于 60 岁的门诊患者比例明显低于 2019 年（全部满足 *P* < 0.0001）。眼部视力较低值低于 5/60 或需要转诊的门诊患者比例明显更高（两者均满足 *P *< 0.0001）。

**结论:**

限制性测量导致门诊就诊率降低，但二级和视力中心的下降幅度小于三级中心。通过远程医疗可轻松获得专业眼科医疗保健服务，且这些中心与社区相对较近，有助于减少医疗服务获取相关障碍。

## Резюме

**Цель:**

Изучить влияние пандемии коронавирусного заболевания (COVID-19) на число амбулаторных посещений офтальмологических учреждений на юге Индии.

**Методы:**

Авторы использовали данные о 7,69 млн амбулаторных посещений в офтальмологические центры первичного (то есть центры зрения), вторичного и третичного звена, принадлежащие сети Aravind Eye Care System, в период с января 2019 года по июнь 2021 года. Сравнивалось количество амбулаторных пациентов, а также их возраст и пол между периодом пандемии и допандемическим периодом в 2019 году во всех центрах, тогда как оценки зрения и офтальмологические обследования сравнивались только среди центров зрения.

**Результаты:**

Во время первой волны пандемии количество амбулаторных посещений в центрах третичного и вторичного звена, а также центрах зрения составило 39% (647 968/1 656 296), 60% (170 934/283 176) и 73% (180 502/246 282) от уровня 2019 года соответственно. Во время второй волны пандемии количество амбулаторных посещений в центрах третичного и вторичного звена, а также центрах зрения составило 54% (385 092/710 949), 73% (88 383/121 739) и 79% (121 993/154 007) от уровня 2019 года соответственно. Доля амбулаторных пациентов женского пола или моложе 20 лет, или старше 60 лет была значительно ниже в течение первой и второй волн пандемии, чем в 2019 году (*P* < 0,0001 для всех центров). Доля амбулаторных пациентов с остротой зрения менее показателя 5/60 или пациентов, которым требовалось направление к специалисту, была значительно выше (*P* < 0,0001 для обеих групп).

**Вывод:**

Ограничительные меры привели к снижению количества амбулаторных посещений, однако снижение количества посещений было меньше в центрах вторичного звена и центрах зрения, нежели в центрах третичного звена. Свободный доступ к специализированной офтальмологической помощи с помощью телемедицины, а также относительная близость этих центров к общинам помогли уменьшить препятствия к получению такой помощи.

## Introduction

Although the coronavirus disease 2019 (COVID-19) pandemic generated tremendous suffering worldwide, it also provided an opportunity to study patient behaviour. During the early stages of the pandemic, many countries adopted stringent measures to contain the disease. In India, emergency health-care services continued but regular outpatient services were suspended and elective surgery deferred. Despite the stepwise reopening of outpatient eye care following expert committee guidelines,^1^ we witnessed a drastic decline in clinic visits and procedures, which reflected travel restrictions, unemployment-related financial challenges and fear of infection. During the acute phase of the pandemic, patient numbers at one tertiary eye care hospital in India fell to a mere 3.5% of the previous year’s figure.^2^^,^^3^ A similar pattern was seen in the United States of America (USA) and was possibly repeated worldwide.^4^ Although the pandemic adversely affected access to eye care, the number of sight-threatening conditions occurring would nevertheless have been expected to remain the same and any delays in care could have led to an increasing number of individuals becoming irreversibly blind or experiencing a reduced quality of life.

In India, hospitals were able to remain open for emergencies and critical care. Secondary and tertiary hospitals belonging to the Aravind Eye Care System network in south India, which handle 4.6 million outpatients per year, were among the few eye hospitals in the country that remained open throughout the pandemic. Ophthalmic care was also provided by private multispecialty hospitals but they were overwhelmed by the inflow of COVID-19 patients. Moreover, most private eye clinics were closed during the acute phases of the pandemic.^2^ Outreach eye camps run by the Aravind Eye Care System, which cater to rural populations, also ceased operating due to the restrictive measurements.^2^^,^^5^ In contrast, the Aravind Eye Care System’s primary eye-care centres, known as vision centres, started to function in a phased manner in accordance with local restrictions. With travel restrictions in force to contain the spread of COVID-19, it seemed logical that patients would prefer to seek care locally.

The aim of this study was to examine the overall impact of travel restrictions, the closure of eye-care facilities, evolving patient preferences and other challenges associated with COVID-19 on the volume and nature of outpatient visits to primary, secondary and tertiary levels of eye care in south India. We hypothesized that patients may have accessed vision centres more often in these unprecedented times by virtue of their proximity to communities. Our analysis compared outpatient attendance at all facilities in the Aravind Eye Care System and the severity of presenting eye conditions at vision centres during the COVID-19 pandemic with data for the preceding year to understand changes in the way patients accessed care.

## Methods

We conducted a cross-sectional study of 87 eye-care centres belonging to the Aravind Eye Care System in the states of Tamil Nadu and Pondicherry in south India: (i) six were tertiary eye care hospitals equipped to cater for the entire spectrum of eye-care services; (ii) six were secondary eye-care hospitals that provide comprehensive eye examinations by ophthalmologists and offer cataract surgery and other minor procedures; and (iii) 75 were primary eye-care centres (i.e. vision centres), which offer in-person examinations by a vision technician (who has skills similar to an optometrist) and teleconsultations with an ophthalmologist at a base hospital. Vision centres can perform refraction, slit-lamp biomicroscopy, applanation tonometry and fundus imaging. Each centre serves a population of 50 000 to 70 000 residing within a radius of 8 to 10 km. Of note, all Aravind Eye Care System facilities are run entirely on a walk-in basis with no appointment system. Thus, patient volumes are a good reflection of health-seeking behaviour. During both the first and second waves of the COVID-19 pandemic, all sites followed recommended COVID-19 protocols for examining patients, which included strict adherence to mask-wearing, social distancing and hand hygiene.^6^

We reviewed the electronic medical records of all consecutive outpatients seen at the facilities between January 2020 and June 2021. To study trends in outpatient behaviour and characteristics, we considered four time periods: (i) the period immediately before the COVID-19 pandemic from 1 January to 23 March 2020 (i.e. the pre-COVID-19 period); (ii) the first wave of the pandemic from 24 March to 31 October 2020; (iii) the period after the first wave from 1 November 2020 to 31 March 2021; and (iv) the second wave from 1 April to 30 June 2021. Table 1 lists the COVID-19 restrictions in place at study sites during different phases of the pandemic. Data for these time periods were compared with data for corresponding periods in 2019, matched by day of the week and month (Table 1). Comparative 2019 data for each vision centre included only data for dates corresponding to those dates in 2020 and 2021 when that centre was open. Vision centres established in 2020 or 2021 were not included in the study. The patients’ demographic characteristics, including age and sex, across all facilities were analysed specifically for the first and second waves of the pandemic. In addition, presenting vision assessments and clinical diagnoses were studied only in vision centres and only during the two waves of the pandemic. Although these two waves spanned 15 months across 2020 and 2021, the comparison period covered only the corresponding days in 2019, the year immediately preceding the COVID-19 pandemic (Table 1).

**Table 1:** Study and data comparison periods and COVID-19 restrictions, study of outpatient eye care during the pandemic, Pondicherry and Tamil Nadu, India, 2019–2021

Study period	Study period dates	COVID-19 restrictions	Dates of data comparison period^a^
Pre-COVID-19	1 Jan 2020 to 23 Mar 2020	None	1 Jan 2019 to 23 Mar 2019
First wave of the COVID-19 pandemic	24 Mar 2020 to 31 Oct 2020	Phase I (24 Mar to 3 May 2020): no private or public transport; phase II (4–17 May 2020): travel in private vehicles allowed with official permission but no public transport; phase III (18–31 May 2020): travel in private vehicles and taxis allowed with official permission but no public transport; phase IV (1–23 Jun 2020): all vehicle types, including mass public transportation and private vehicles, allowed with official permission; phase V (24 Jun to 31 Aug 2020): as phase III; Post-restrictions phase (1 Sep to 31 Oct 2020): no travel restrictions	24 Mar 2019 to 31 Oct 2019
After the first wave	1 Nov 2020 to 31 Mar 2021	No travel restrictions	1 Nov 2019 to 31 Dec 2019 and 1 Jan 2019 to 31 Mar 2019
Second wave of the pandemic	1 Apr 2021 to 30 Jun 2021	Phase I (1 Apr 2021 to 9 May 2021): no travel restrictions; phase II (10 May 2021 to 30 Jun 2021): private vehicles allowed with official permission but no public transport	1 Apr 2019 to 30 Jun 2019

COVID-19: coronavirus disease 2019.

^a^ Data for 1 January to 30 June 2019 were used twice to compare the first and second waves of the pandemic, respectively, with the most recent year without COVID-19 (i.e. 2019), during which there were 3 793 904 unique outpatient visits to Aravind Eye Care System facilities.

Variables extracted from medical records for each visit included the date of the visit and the patient’s age and sex. For patients attending vision centres, additional information was obtained on visual acuity at presentation and on the diagnosis for the worse eye during the first and second waves and the respective comparison periods. If there was more than one diagnosis, the diagnosis that most threatened vision was considered the primary diagnosis. For example, if a patient had an immature cataract and retinal detachment, the primary diagnosis was retinal detachment.

To understand changes in patient behaviour during the pandemic, we contacted a random subset of patients by phone and, after obtaining informed consent orally, asked them why they delayed or missed consultations. Similarly, we spoke to patients who attended hospitals to determine what motivated their visit during the COVID-19 pandemic.

We followed the tenets of the Declaration of Helsinki and obtained ethical clearance from the institutional ethics committee of the Aravind Eye Hospital in Pondicherry.

### Statistical analysis

Data were saved on Excel (Microsoft Corporation, Redmond, USA) and analysed using Stata v.14.0 (StataCorp LLC, College Station, USA). We compared differences in variables between the pandemic and pre-pandemic periods using two-sample proportion tests. A *P* value less than 0.05 was considered statistically significant. 

## Results

We obtained data on 7.69 million outpatient visits to 87 Aravind Eye Care System centres between 1 January 2019 and 30 June 2021 (Table 2). During the pre-COVID-19 period, the number of outpatient visits was 3 to 8% higher across all facilities than during the comparison period. When the first wave of the pandemic occurred, the outpatient volume decreased across all eye-care levels: compared with 2019, the volume during the first wave was 73% (180 502/246 282) at vision centres, 60% (170 934/283 176) at secondary care centres and 39% (647 968/1 656 296) at tertiary care centres. After the first wave, outpatient visits bounced back rapidly across all levels of eye care. During the second wave, visits to vision centres again held up better than visits to secondary or tertiary centres: compared with 2019, the volume during the second wave was 79% (121 993/154 007) at vision centres, 73% (88 383/121 739) at secondary care centres and 54% (385 092/710 949) at tertiary care centres. 

**Table 2:** Outpatient visits to Aravind Eye Care System facilities before and during the COVID-19 pandemic waves, Pondicherry and Tamil Nadu, India, 2019–2021

Study period^a^	No. outpatient visits							Study period visits as a proportion of comparison period visits (significance of difference between the periods)		
	Study period in 2020 and 2021			Comparison period in 2019^b,c^				% (*P* value)^d^		
	Tertiary care hospitals	Secondary care hospitals	Vision centres^e^	Tertiary care hospitals	Secondary care hospitals	Vision centres^e^		Tertiary care hospitals	Secondary care hospitals	Vision centres^e^
**Pre-COVID-19**	572 376	101 501	159 122	553 475	96 949	147 261		103 (< 0.0001)	105 (0.54)	108 (< 0.0001)
**First wave of the COVID-19 pandemic**	647 968	170 934	180 502	1 656 296	283 176	246 282		39 (< 0.0001)	60 (< 0.0001)	73 (< 0.0001)
**After the first wave**	1 037 133	187 089	247 006	1 046 539	180 841	272 754		99 (< 0.0001)	103 (< 0.0001)	91 (< 0.0001)
**Second wave of the pandemic**	385 092	88 383	121 993	710 949	121 739	154 007		54 (< 0.0001)	73 (< 0.0001)	79 (< 0.0001)
**Total**	**2 642 569**	**547 907**	**708 623**	**3 967 259**	**682 705**	**820 304**		**67 (NA)**	**80 (NA)**	**86 (NA)**

COVID-19: coronavirus disease 2019; NA: not applicable.

^a^ The pre-COVID-19 period was from 1 January 2020 to 23 March 2020; the first wave of the COVID-19 pandemic was from 24 March 2020 to 31 October 2020; the period after the first wave was from 1 November 2020 to 31 March 2021; and the second wave of the pandemic was from 1 April 2021 to 30 June 2021 (Table 1).

^b^ For the comparison period, dates in the study period were matched with dates in 2019, the last year before the pandemic (Table 1).

^c^ The 1 674 364 outpatient visits made between January and June 2019 were used twice for comparisons. Consequently, this figure was deducted when the total number of unique outpatient visits was calculated.

^d^ *P* values were derived using the two-sample proportion test.

^e^ Vision centres are primary care centres.

**Fig. 1:**
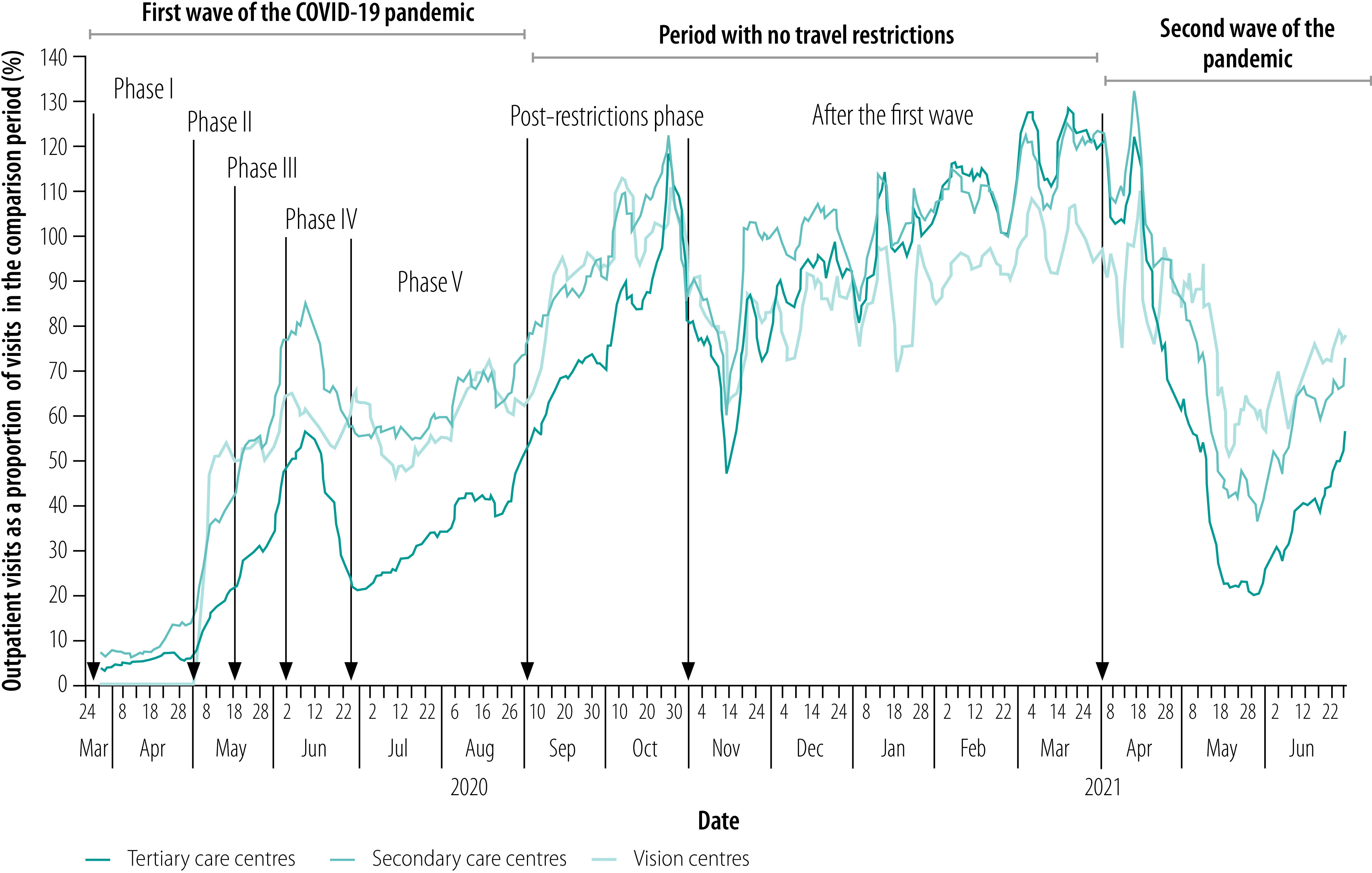
Outpatient visits to Aravind Eye Care System vision facilities during COVID-19 pandemic waves, Pondicherry and Tamil Nadu, India, March 2020 to June 2021

Fig. 1 shows a moving average of the number of outpatient visits between March 2020 and June 2021 as a proportion of the number during the comparison period for each eye care facility level. During phase I in the first wave of the pandemic from 24 March 2020 to 3 May 2020, when there was no transport (Table 1), the outpatient volume at tertiary and secondary hospitals was only 5.3% (16 019/300 590) and 9.5% (4825/50 656) of 2019 volumes, respectively; vision centres were closed. During phases II, III and IV in the first wave, outpatient volumes gradually increased across all levels of care. In general, the volume increased more rapidly at vision centres than at tertiary or secondary centres, except in phase IV, when the volume at vision centres during the study period as a proportion of that during the 2019 comparison period fell to 58% (18 722/32 321) overall because several vision centres were located in newly declared containment zones where movement was restricted. Between 1 September and 31 October 2020, when all travel restrictions were lifted (Table 1), outpatient volumes increased steadily across all levels of care. During the second wave, from 10 May to 30 June 2021, vision centres again performed better than tertiary or secondary care centres.

COVID-19: coronavirus disease 2019.

Notes: The graph shows the six-day moving average of outpatient visits during the pandemic in 2020 and 2021 as a percentage of the number of outpatient visits during the comparison period in 2019. For the comparison period, dates in the study period were matched with dates in 2019, the last year before the pandemic (Table 1). Travel restrictions during the first wave of the pandemic were applied in five phases (for details, see Table 1). Vision centres were closed during phase I. Although the second wave started on 1 April 2021, travel restrictions were not imposed until 10 May 2021.

Fig. 1 shows that the vision centres witnessed a rapid increase in patient numbers when they reopened after travel restrictions were gradually lifted at the end of phase I in the first wave: they reached 49% (3384/6926) of the 2019 volume within a week of reopening. In contrast, tertiary eye-care centres reached 44% (81 903/185 483) of the 2019 volume only after about 6 weeks. In addition, when all travel restrictions had been lifted after phase V, tertiary centres had only 66% (150 540/229 013) of the previous year’s volume on average, whereas vision centres and secondary centres recovered to 88% (47 957/54 714) and 86% (32 833/37 981), respectively. After the first wave, a rapid bounce back was seen across all levels of eye care. This was followed by another drastic decline in patient volume during the restrictions in the second wave, although vision centres performed better.

Table 3 shows the age and sex distributions, respectively, of outpatients visiting vision centres and secondary and tertiary care centres during the first and second waves of the COVID-19 pandemic and during the comparison periods. Though the absolute number of visits was lower during the first and second waves than during 2019 overall, the proportion of all patients who were aged 21–60 years was significantly higher during the pandemic across all facilities (*P* < 0.0001 for all). Correspondingly, the proportion of all patients who were aged 61 years and older or 20 years and younger was lower during the pandemic across all levels of eye care (*P* < 0.001 for all). In addition, the proportion of patients who were female was significantly lower during the pandemic compared to 2019 across all facilities (*P* < 0.0001 for all).

**Table 3:** Outpatient visits to Aravind Eye Care System facilities during and before COVID-19 pandemic waves, by age, Pondicherry and Tamil Nadu, India, 2019–2021

Outpatients’ characteristic	Tertiary care centres				Secondary care centres				Vision centres^a^		
	No. outpatient visits (%)		*P* value^b^		No. outpatient visits (%)		*P* value^b^		No. outpatient visits (%)		*P* value^b^
	Pandemic period^c^ (*n* = 1 033 060)	Comparison period^d^ (*n* = 2 418 084)			Pandemic period^c^ (*n* = 259 317)	Comparison period^d^ (*n* = 413 690)			Pandemic period^c^ (*n* = 302 495)	Comparison period^d^ (*n* = 414 100)	
**Age, years**											
≤ 20	107 361 (10.39)	326 370 (13.50)	< 0.0001		25 848 (9.97)	53 146 (12.85)	< 0.0001		45 669 (15.10)	74 554 (18.00)	< 0.0001
21–40	188 899 (18.29)	377 869 (15.63)	< 0.0001		52 371 (20.20)	70 838 (17.12)	< 0.0001		85 661 (28.32)	110 058 (26.58)	< 0.0001
41–60	427 611 (41.39)	911 047 (37.68)	< 0.0001		104 391 (40.26)	150 873 (36.47)	< 0.0001		107 995 (35.70)	140 321 (33.89)	< 0.0001
61–75	276 277 (26.74)	704 733 (29.14)	< 0.0001		67 320 (25.96)	119 536 (28.90)	< 0.0001		55 521 (18.35)	77 331 (18.67)	< 0.0001
> 75	32 912 (3.19)	98 065 (4.06)	< 0.0001		9 387 (3.61)	19 297 (4.66)	< 0.0001		7 649 (2.53)	11 836 (2.86)	0.0006
**Sex**											
Female	483 898 (46.84)	1 183 564 (48.95)	< 0.0001		133 081 (51.32)	226 891 (54.85)	< 0.0001		146 623 (48.47)	209 657 (50.63)	< 0.0001
Male	549 162 (53.16)	1 234 520 (51.05)	< 0.0001		126 236 (48.68)	186 799 (45.15)	< 0.0001		155 872 (51.53)	204 443 (49.37)	< 0.0001

COVID-19: coronavirus disease 2019.

^a^ Vision centres are primary care centres.

^b^ *P* values were derived using the two-sample proportion test.

^c^ The pandemic period comprised two waves of the pandemic from 24 March 2020 to 31 October 2020 and from 1 April 2021 to 30 June 2021, respectively.

^d^ The corresponding comparison periods were from 24 March 2019 to 31 October 2019 and from 1 April 2019 to 30 June 2019, respectively.

Table 4 and Table 5 show the distributions of visual acuity and ophthalmic diagnosis at presentation, respectively, recorded on outpatient visits to vision centres during the first and second waves of the pandemic and during the comparison periods in 2019. The proportion of patients who presented with a visual acuity of 5/60 to 3/60 or of less than 3/60 in the worse eye was significantly higher during the pandemic period than during 2019 (*P* < 0.0001 for both), though the absolute number of visits was smaller (Table 4). In addition, there was a substantial shift in the distribution of diagnoses between 2019 and the pandemic period (Table 5). The proportion of diagnoses that required a referral to a tertiary care hospital (e.g. lens-induced glaucoma, cataract, corneal infection, ocular trauma, uveal disease and retinal disease such as diabetic retinopathy and vein occlusion) was significantly higher during the pandemic (*P* < 0.0001 for all). In contrast, the proportion of diagnoses of glaucoma (other than lens-induced glaucoma) or refractive error was significantly lower (*P* < 0.0001 for both).

**Table 4:** Outpatient visits to Aravind Eye Care System vision centres^a^ during and before COVID-19 pandemic waves, by visual acuity, Pondicherry and Tamil Nadu, India, 2019–2021

Visual acuity in worse eye at presentation	No. outpatient visits (%)		*P* value^b^
	Pandemic period^c^ (*n* = 280 592)^e^	Comparison period^d^ (*n* = 387 906)^e^	
≥ 6/18	198 515 (70.75)	276 722 (71.34)	< 0.0001
6/24–6/60	50 707 (18.07)	73 309 (18.90)	< 0.0001
5/60–3/60	17 137 (6.11)	20 640 (5.32)	< 0.0001
< 3/60	14 233 (5.07)	17 235 (4.44)	< 0.0001

COVID-19: coronavirus disease 2019.

^a^ Vision centres are primary care centres.

^b^ *P* values were derived using the two-sample proportion test.

^c^ The pandemic period comprised the first two waves of the pandemic from 24 March 2020 to 31 October 2020 and from 1 April 2021 to 30 June 2021, respectively.

^d^ The corresponding comparison periods were from 24 March 2019 to 31 October 2019 and from 1 April 2019 to 30 June 2019, respectively.

^e^ The total numbers of outpatient visits differ from the corresponding totals for vision centres shown in Table 3 because visual acuity was not documented in some case records.

**Table 5:** Outpatient visits to Aravind Eye Care System vision centres^a^ during and before COVID-19 pandemic waves, by diagnosis, Pondicherry and Tamil Nadu, India, 2019–2021

Diagnosis for worse eye at presentation	No. outpatient visits (%)		*P* value^b^
	Pandemic period^c^ (*n* = 281 612)^e^	Comparison period^d^ (*n* = 404 515)^e^	
Cataract	60 927 (21.64)	79 718 (19.71)	< 0.0001
Corneal infection	952 (0.34)	446 (0.11)	< 0.0001
Glaucoma	2 241 (0.80)	4 037 (1.00)	< 0.0001
Ocular trauma	10 343 (3.67)	12 069 (2.98)	< 0.0001
Lens-induced glaucoma	71 (0.03)	5 (< 0.01)	< 0.0001
Neuro-ophthalmic condition	283 (0.10)	372 (0.09)	0.26
Refractive error	39 963 (14.19)	60 824 (15.04)	< 0.0001
Retinal detachment	91 (0.03)	114 (0.03)	0.33
Retinal disease	1 914 (0.68)	2 363 (0.58)	< 0.0001
Uveal disease	1 954 (0.69)	1 951 (0.48)	< 0.0001
Other diagnosis	162 873 (57.84)	242 616 (59.98)	< 0.0001

COVID-19: coronavirus disease 2019.

^a^ Vision centres are primary care centres.

^b^ *P* values were derived using the two-sample proportion test.

^c^ The pandemic period comprised the first two waves of the pandemic from 24 March 2020 to 31 October 2020 and from 1 April 2021 to 30 June 2021, respectively.

^d^ The corresponding comparison periods were from 24 March 2019 to 31 October 2019 and from 1 April 2019 to 30 June 2019, respectively.

^e^ The total numbers of outpatient visits differ from the corresponding totals for vision centres in Table 3 because the diagnosis was not documented in some case records.

In the phone survey of a random subset of patients who were asked why they did not attend their regular follow-up, 70% (329/470) mentioned travel restrictions, 35% (164/470) feared getting infected, 13% (61/470) had financial problems, 8.9% (42/470) did not have an escort, 8.7% (41/470) did not feel it was necessary and 17% (79/470) had a consultation elsewhere. These responses were not mutually exclusive. Individuals who attended hospital during the active phase of the pandemic said they sought care because they either were afraid of going blind, had worsening symptoms or anticipated future travel restrictions.

## Discussion

The COVID-19 pandemic provided us with a unique opportunity to study how ease of access affects outpatient visits to primary, secondary and tertiary eye care facilities. Our study, which involved a large number of patients, found that vision centres experienced a smaller decline in outpatient numbers during both waves of the pandemic than secondary or tertiary eye hospitals. These findings reinforce the importance of ease of access for the utilization of care, particularly in underserved and rural communities.

The significant decline in patient numbers we observed during the pandemic was very different from normal fluctuations. In previous years, the patient volume grew consistently and gradually at an annual rate of 2 to 5% across all levels of eye care, with higher growth rates in newly established hospitals and vision centres. Nearly 40% of patients presenting to the Aravind Eye Care System’s tertiary hospitals travelled over 100 km, largely by public bus, with an average travel time of 4 hours.^7^ In contrast, travel to secondary hospitals and vision centres generally took less time, which made them easier to access, especially during the pandemic. Additionally, these facilities tended to have shorter turnaround times and the real-time teleconsultations available at vision centres eliminated the potential risk of exposure to infection during travel. 

Our results align with those of previous studies into the effect of the COVID-19 pandemic on patient care across all health-care facilities.^8^^–^^14^ During phase I in the first wave, when all vision centres were closed, outpatient volumes in tertiary and secondary centres were only 5.3% and 9.5% of corresponding volumes in 2019, respectively. The decline occurred despite our efforts to make the public aware through social media and newspapers that hospitals continued to function with the necessary safety precautions in place. During both waves of the pandemic, staff were assigned to reminding patients about pending care and especially about urgent or emergent care. We also helped patients get the mandatory passes needed to travel to hospital. 

When we investigated the age profile of outpatients, we observed that the proportion who were dependent on others for their care (i.e. those aged under 20 years and those aged over 60 years) was lower during the pandemic period than in 2019. A previous study has shown that the proportion of children and adolescents visiting ophthalmology emergency departments decreased from 10 to 5.3% during the early phases of the COVID-19 pandemic.^13^ Possible reasons for this trend include: (i) fear of infection among adolescents and elderly people; (ii) patients needing someone to escort them; and (iii) comorbid conditions and decreased mobility among the elderly.^14^ Our study also demonstrated that the proportion of female patients decreased significantly across all eye-care levels during the pandemic relative to the previous year. A similar sex bias during the pandemic has been reported in previous studies.^3^^,^^13^ Possible reasons are: (i) male family members being prioritized; (ii) female family members having more family obligations; and (iii) female family members’ perceived role as caregivers.^15^ A sex bias in the utilization of health care in general had been observed in China and India before the COVID-19 pandemic.^15^^–^^17^

Telemedicine became increasingly important during the pandemic for both health care as a whole and ophthalmology.^18^^–^^20^ At our vision centres, routine tele-ophthalmic consultation was advantageous for both health-care providers and patients: access to care improved and the need for travel, with its associated costs and risk of exposure to the virus, decreased. In recognition of the importance of ease of access, we are now systematically shifting care to the local level (e.g. to vision centres), where appropriate. For example, over 55% of patients who have undergone cataract surgery are now seen at the nearest vision centre for postoperative follow-up on day 1. The decentralization of care has also reduced the carbon footprint associated with travel. In addition, we are increasing the scope of telemedicine at our vision centres by integrating the telemedicine process into electronic medical records and facilitating consultations in some subspecialities, which will make care at vision centres more comprehensive. We believe that the processes triggered by COVID-19 will bring lasting improvements. The effective utilization of telemedicine in primary care has already produced highly encouraging results. Moreover, patients’ real-time teleconsultations with ophthalmologists provide opportunities for continuing clinical education for vision technicians at vision centres, which can reinforce their skill development. Still, continuing training of staff at secondary and primary care levels is important for improving the quality of diagnosis and referrals. This model of augmenting primary eye-care centres with telemedicine has the potential to revolutionize the entire health-care system.

Our study has several strengths: (i) it involved a large data set of 7.69 million outpatient visits across all levels of eye care; (ii) it covered both the first and second waves of the COVID-19 pandemic; and (iii) it compared pandemic and pre-pandemic periods. Additionally, the Aravind Eye Care System does not have an appointments system, which eliminates provider-related biases and, thus, ensures that data reflect the natural change in patient numbers. Our study was limited by its retrospective nature and by a lack of data on presenting morbidity patterns at tertiary and secondary eye-care hospitals. Moreover, we did not analyse compliance with referrals from vision centres to secondary and tertiary hospitals. Finally, our study was done in south India, which could limit the generalizability of our results because pandemic restrictions varied widely across different parts of the world.

In conclusion, we believe the trend we observed of patients seeking care for both emergency and non-emergency conditions closer to home will be relevant after the pandemic, particularly now that specialist consultations are available via telemedicine. This trend may be especially important for countries with large rural and semi-urban populations, where access to health care is difficult. Our study’s findings suggest that primary eye-care centres and secondary eye-care hospitals can continue to provide care even in crises such as the COVID-19 pandemic by reducing barriers to access. With technology evolving rapidly and internet coverage increasing, vision centres offering artificial intelligence-assisted evaluations could soon become a reality, which would substantially improve the quality of care. As the investment and gestation period needed for establishing vision centres are small, a rapid scale-up should be possible. More vision centres coupled with well-monitored training of personnel would help preserve vision among disadvantaged people, even after the present pandemic. Health-care systems must continue to adapt to the evolving needs and preferences of patients, to advances in technology and to the sweeping restrictions periodically introduced during health crises.
